# Immunotherapy Landscape of Advanced Clear Cell Renal Cell Carcinoma: Targeting the Cancer-Immunity Cycle and Future Perspectives

**DOI:** 10.3390/biomedicines14061181

**Published:** 2026-05-22

**Authors:** Xuanyu Jin, Junkai Yang, Daojia Miao, Wei Xiong, Zhiyong Xiong

**Affiliations:** 1 Department of Urology, Union Hospital, Tongji Medical College, Huazhong University of Science and Technology, Wuhan 430022, China; 19352888638@163.com (X.J.); yjkjoshua@163.com (J.Y.); 2 Institute of Urology, Union Hospital, Tongji Medical College, Huazhong University of Science and Technology, Wuhan 430022, China; 3 Department of Nephrology, Union Hospital, Tongji Medical College, Huazhong University of Science and Technology, Wuhan 430022, China

**Keywords:** renal cell carcinoma, immunotherapy, cancer-immunity cycle, immune checkpoint inhibitors, personalized therapy

## Abstract

Renal cell carcinoma (RCC) is a predominant malignancy of the urinary system, with clear cell renal cell carcinoma (ccRCC) representing 75–85% of clinical cases. Since the early stages are often asymptomatic, nearly 30% of patients present with metastases at diagnosis, which significantly complicates the prognosis. The diverse mechanisms and clinical indications of current strategies, despite recent breakthroughs in immunotherapy, pose a major challenge for systematic application. This review employs the cancer-immunity cycle as a framework to evaluate four critical steps: antigen presentation, T-cell activation, reversal of exhaustion, and immune evasion in the tumor microenvironment. We introduce the major immunotherapy strategies in RCC in this cycle and summarize their clinical position. Combining immune checkpoint inhibitors (ICIs) with tyrosine kinase inhibitors (TKI) has redefined the first-line standard for advanced RCC by addressing both T-cell infiltration barriers and functional suppression. Standalone approaches such as tumor vaccines and cytokines in contrast have shown limited efficacy in advanced settings. In this context, we further propose emerging research directions, such as individualized immunotherapy and multi-target blockade, and point out the relevant biomarkers, offering an integrated perspective of the RCC immune landscape and providing insights for both clinical practice and future research.

## 1. Introduction

### 1.1. Epidemiology and Therapeutic Challenges of RCC

Renal cell carcinoma (RCC) is a type of cancer that begins in the renal tubular epithelial cells, representing more than 90% of kidney cancers, and is the fifteenth most frequent cancer worldwide [1]. Clear cell renal cell carcinoma (ccRCC) is the most widespread histological subtype of RCC, comprising 75–85% of all kidney cancers, and is the primary pathological type in metastatic RCC [2]. In 2024, an estimated 81,610 new kidney cancer cases are expected in the United States, making up 4.1% of all new cancer diagnoses; meanwhile, there will be 14,390 related deaths, making up 2.4% of cancer-related mortalities [3], with a higher incidence in males. While early-stage RCC can be cured through surgery, approximately 30% of patients already present with metastases at the time of diagnosis, and 30–40% of patients will experience recurrence after surgery, with 50% of these cases leading to distant metastases, resulting in poor prognosis [4,5]. For advanced RCC, traditional treatments, including tyrosine kinase inhibitors (TKIs), chemotherapy, and radiation therapy, can delay disease progression but show limited complete response rates and durability of efficacy [6]. Notably, RCC generally exhibits high immunogenicity, characterized by substantial infiltration of immune cells within the tumor microenvironment, and exhibits a certain degree of sensitivity to immune modulation. This biological feature provides an important theoretical foundation for immunotherapy. Recent studies have shown that immunotherapy can lead to long-lasting clinical responses in certain patients and significantly improve long-term survival outcomes [3,7].

### 1.2. Overview of Tumor Immunotherapy and the Rise and Development of Immunotherapy in RCC

With breakthroughs in immuno-oncology (IO) research, tumor immunotherapy has rapidly evolved into a promising and fast-growing domain in oncology. This therapy mainly works by activating the host’s inherent adaptive and innate immune systems to identify and eliminate tumor cells, thus possessing specificity and persistence that traditional chemotherapy does not have [8]. Its origin can be traced back to the accidental discovery of Coley toxin in the late 19th century, while the modern immunotherapy system is based on in-depth research on the regulatory mechanism of T-cell activation and immune checkpoint molecules [2]. In 2013, Chen and Mellman systematically outlined the cancer-immunity cycle theory [9], summarizing the anti-tumor immune response into seven sequential biological processes: tumor antigen release, antigen presentation and T-cell activation, T-cell activation and expansion, T-cell migration to the tumor, T-cell infiltration into the tumor, T-cell-specific recognition of tumor cells, and finally the elimination of tumor cells [9]. Dysfunction in any step of this cycle can result in immune evasion by the tumor.

As an immunogenic tumor, RCC’s immunotherapy development has essentially involved the progressive development of interventions targeting key steps in the cancer-immunity cycle. From the early stage of using cytokines to non-specifically enhance T-cell proliferation to the precise reversal of T-cell exhaustion by immune checkpoint inhibitors, and then to the improvement of the immunosuppressive microenvironment with anti-angiogenic drugs to promote T-cell infiltration, the progress of immunotherapy for renal cancer is actually an exploration of the regulatory ability of the cancer-immune cycle.

**Figure 1:**
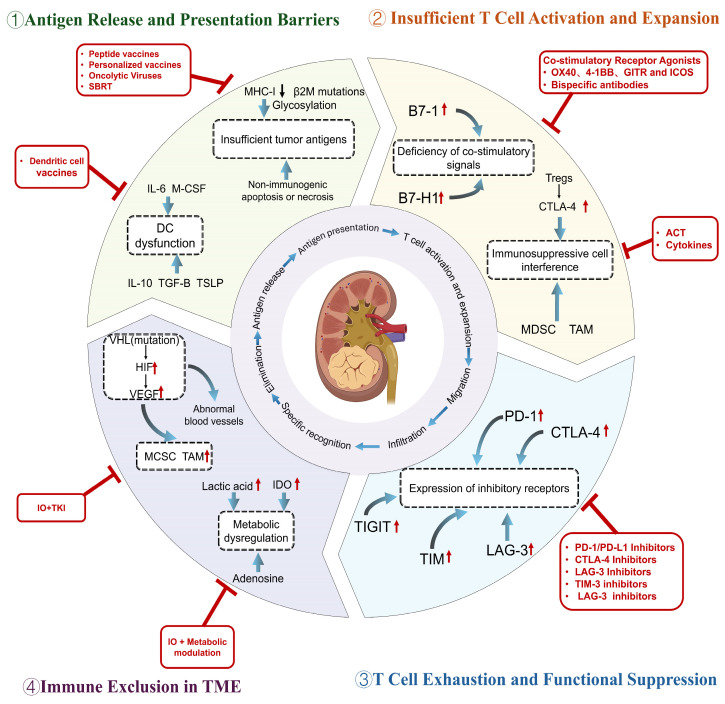
Panoramic view of RCC immunotherapy based on the cancer-immunity cycle. Schematic illustration depicting the cancer-immunity cycle in renal cell carcinoma (RCC), with sequential steps of antitumor immunity (antigen release, antigen presentation, T-cell activation and expansion, migration, infiltration, specific recognition, and elimination). The central kidney is surrounded by four quadrants representing distinct barriers to effective antitumor immunity: (1) antigen release and presentation barriers; (2) insufficient T-cell activation and expansion; (3) T-cell exhaustion and functional suppression; and (4) immune exclusion in the tumor microenvironment. Red boxes indicate corresponding therapeutic interventions; dashed boxes denote pathological mechanisms. Upward arrows indicate upregulation and downward arrows indicate downregulation. (The figure was created using PowerPoint and Biorender.com.) Abbreviations: ACT, adoptive cell transfer; CTLA-4, cytotoxic T-lymphocyte-associated protein 4; HIF, hypoxia-inducible factor; DC, dendritic cell; ICOS, inducible T-cell co-stimulator; IDO, indoleamine 2,3-dioxygenase; IO, immuno-oncology; MHC-I, major histocompatibility complex class I; MDSC, myeloid-derived suppressor cell; LAG-3, lymphocyte activation gene-3; PD-1, programmed cell death protein 1; PD-L1, PD-1 ligand 1; SBRT, stereotactic body radiation therapy; TIM, T-cell immunoglobulin and mucin-domain containing; TIGIT, T-cell immunoreceptor with Ig and ITIM domains; TAM, tumor-associated macrophage; TKI, tyrosine kinase inhibitor; VEGF, vascular endothelial growth factor; Treg, regulatory T-cell; TME, tumor microenvironment; VHL, von Hippel–Lindau.

This article takes the cancer-immunity cycle as the central framework and systematically reviews the immunotherapeutic strategies developed for the four key steps in RCC (Figure 1). It elaborates on the mechanisms of action, representative drugs, and clinical research status of various therapeutic approaches, while also summarizing the current challenges and clinical dilemmas faced in RCC immunotherapy, aiming to provide an integrated perspective on the current state of immunotherapy in RCC. Given that most available immunotherapy evidence in RCC derives from advanced clear cell RCC (ccRCC), this review primarily focuses on systemic immunotherapeutic strategies in advanced/metastatic ccRCC. Non-clear cell histologies, localized disease, and perioperative immunotherapy are discussed only briefly where relevant.

## 2. Immunotherapy Strategies Targeting Different Phases of the Cancer-Immunity Cycle

### 2.1. Antigen Release and Presentation Barriers

#### 2.1.1. Mechanistic Basis

An anti-tumor immune response that is effective starts with the release, capture, and presentation of antigens from tumors. The proper functioning of this process is essential for initiating subsequent T-cell responses. However, in RCC, multiple functional impairments often occur in the antigen release and presentation phase, limiting the initiation of T-cell responses and promoting immune evasion. Among the most widely studied mechanisms are dendritic cell (DC) dysfunction and insufficient release and presentation of tumor-specific antigens.

As a key link between the innate and adaptive immune systems, DCs are central to the regulation of T-cell responses that are specific to antigens [10]. The initial step of the normal specific immune response is that an antigen released by tumor cells after death is captured by DCs, processed and presented to T cells through major histocompatibility complex molecules [11,12]. In RCC patients, however, this process is frequently disrupted by several factors. Research indicates that the frequency of myeloid dendritic cells and plasmacytoid dendritic cells in the peripheral blood of patients with renal cell carcinoma is significantly lower than that in healthy people. Most of the dendritic cells infiltrated into the tumor tissue show an immature phenotype, and cannot effectively differentiate into mature dendritic cells, resulting in a decline in antigen uptake, processing and presentation ability [13]. The mechanisms contributing to this dysfunction mainly include: tumors inhibit T-cell priming by inducing the differentiation of monocytes into macrophages rather than DCs via IL-6 and M-CSF (macrophage-stimulating factor); carcinoembryonic antigens such as MUC1 (mucin 1) are endocytosed and retained in early endosomes, where they cannot be efficiently processed and presented; and cytokines such as IL-10, TGF-β (transforming growth factor-β), and TSLP (Thymic Stromal Lymphopoietin) secreted by tumors inhibit DC maturation, thereby inducing antigen-specific anergy or generating tumor-promoting DC [14]. Furthermore, immunosuppressive factors in the tumor microenvironment restrict DC maturation and migration to lymph nodes, leading to a reduction in DC frequency and functionality in peripheral blood and tumor tissue, ultimately weakening T-cell responses [13].

Tumor cells themselves can also evade antigen presentation through various mechanisms. RCC cells commonly suppress MHC-I molecule expression, resulting in lower tumor antigen presentation on the cell surface [15]. The β2-microglobulin gene, when mutated or deleted, can directly result in MHC-I assembly defects, contributing to primary resistance in RCC immunotherapy. Recent studies have shown that glycosylation modifications can also serve as a novel immune checkpoint contributing to immune evasion. On CD8+ T cells, sialylated CD56 molecules can connect with SIGLEC-7, which decreases interferon-γ and tumor necrosis factor-α production and induces apoptosis in T cells [16].

Additionally, the type of tumor cell death affects antigen immunogenicity. Immunogenic cell death releases damage-associated molecular patterns (DAMPs) like high-mobility group box 1 (HMGB1), ATP, and calreticulin, which serve as danger signals to aid in DC maturation and cross-presentation of antigens [17]. However, in the RCC tumor microenvironment, tumor cells typically undergo non-immunogenic apoptosis or necrosis, failing to effectively release these danger signals, leading to inefficient antigen presentation [18,19]. It should be pointed out that among the mechanisms discussed in this section, DC dysfunction and MHC-I downregulation are well-documented in humans and have been translated into a clinical target, while the specific roles of glycosylation-mediated immune checkpoints and non-immunogenic cell death are mainly supported by preclinical and in vitro evidence and need further clinical verification.

#### 2.1.2. Tumor Vaccines

To address the issue of insufficient endogenous tumor antigen release, the most direct intervention strategy is to provide tumor antigens exogenously. Tumor vaccines are based on this idea, where pre-prepared tumor antigens are introduced into the body, captured by dendritic cells, and presented to T cells. Tumor vaccines encompass dendritic cell vaccines, peptide vaccines, and personalized vaccine strategies.

Peptide vaccines are composed of specific tumor-associated antigen peptides, which are designed to bypass the obstacles of endogenous antigen processing in tumor cells and directly provide antigen peptides that can be ingested and presented. After intradermal or subcutaneous injection, local DCs take up these peptides and migrate to regional lymph nodes, where they activate specific T-cell responses [20]. IMA901 is a representative peptide vaccine for RCC, composed of nine HLA-A02-restricted peptides and one HLA-DR-restricted peptide, originating from antigens that are overexpressed in RCC. The vaccine is administered with GM-CSF as an adjuvant, and a single low dose of cyclophosphamide is given before the first dose to deplete regulatory T cells [21]. Clinical studies confirmed the immunogenicity of IMA901, but also revealed the limitations of peptide vaccines in advanced RCC. phase I/II studies showed that in some patients, IMA901 was able to induce specific T-cell responses, while immune reactivity against multiple peptides was associated with improved disease control. In a Phase II randomized study, the cyclophosphamide pre-treatment group showed a trend of improved median overall survival, but this difference was not significant. In patients who had previously received cytokine therapy, the disease control rate was 31%, while it was only 14% in those who had received tyrosine kinase inhibitors. In terms of safety, IMA901 was generally well-tolerated [21]. The phase III IMPRINT trial enrolled 340 HLA-A02-positive metastatic RCC patients to assess the efficacy of IMA901 combined with sunitinib versus sunitinib alone as a first-line treatment [22]. However, the study failed to demonstrate that the combination treatment improved overall survival, leading to the termination of the clinical development of IMA901 in advanced RCC. This failure suggests that in an immunosuppressive tumor microenvironment, enhanced antigen infusion alone cannot overcome subsequent immunosuppressive components (such as T-cell depletion and immune rejection), thereby limiting efficacy [21].

The mechanism of dendritic cell vaccines is primarily to bypass the problem of defective dendritic cell function in vivo by loading patient-derived dendritic cells with tumor antigens in vitro and then transfusing them back into the body. In a phase II study involving patients with metastatic RCC, AGS-003, a dendritic cell vaccine loaded with autologous tumor RNA and transfected with CD40L RNA, was administered in combination with sunitinib, achieving a median overall survival of 30.2 months [23], with no unexpected toxicity beyond the known safety profile of sunitinib. However, the subsequent phase III ADAPT study was terminated prematurely due to ineffective interim analysis. This suggests to us that the application of DC vaccines in advanced renal cancer still needs to overcome the challenge of immunosuppression in the tumor microenvironment. Despite this, the application of dendritic cell vaccine in renal cell carcinoma still shows certain potential and is under continuous exploration. In general, although DC vaccine can improve the process of antigen presentation, its efficacy is still limited in the absence of synchronous regulation of the immunosuppressive microenvironment.

In recent years, personalized vaccines based on tumor neoantigens have gradually become a research hotspot. Neoantigens are specific antigens that are generated by genomic mutations in tumor cells and are therefore not present in normal tissues, thus eliciting highly specific T-cell responses and causing less autoimmune toxicity [24]. Personalized neoantigen vaccines have shown clinical value in tumors characterized by a high mutational barrier like melanoma, but whether they are effective in RCC, a tumor with a lower mutational burden, has been a subject of debate. In 2020, Reustle et al. identified tumor-specific, overexpressed HLA-presented peptides in RCC through integrated omics analysis, providing an important foundation for vaccine target selection [25]. A phase I clinical trial reported in *Nature* in 2025 (NCT02950766) assessed the tolerability and immunogenicity of personalized neoantigen vaccines in nine high-risk RCC patients [26]. These patients, after undergoing surgical resection, were vaccinated with personalized vaccines targeting their tumor-driving gene mutations. With a median follow-up of 40.2 months, no tumor recurrence was found in all patients and no vaccine-related serious adverse events were reported. The vaccine successfully induced specific T-cell immune responses against common RCC mutations such as VHL, PBRM1, and BAP1, with these T cells persisting long-term in the body. The encouraging immunological outcomes observed in this study may be largely attributed to targeting tumor-specific neoantigens rather than self-tolerant antigens, fundamentally enhancing antigen immunogenicity. However, this finding is based on a single phase I trial involving only nine highly selected, post-surgical patients, and whether this approach can be extended to larger populations or advanced disease settings remains to be determined.

Among the three types of vaccines discussed, personalized neoantigen vaccines currently show the clearest potential, while phase III studies of peptide and DC vaccines have both failed. However, this observation is derived from a small adjuvant trial and should be regarded as hypothesis-generating rather than practice-confirming at this stage. The reasons for these failures, aside from the inability to overcome other barriers in the cycle when used alone, may also relate to antigen selection and the choice of patient populations. The antigens used in IMA901 are likewise present in certain normal tissues, limiting the induction of high-affinity T-cell responses due to central immune tolerance, while personalized vaccines target neoantigens that are not subject to immune tolerance. Moreover, IMA901 and AGS-003 were tested in advanced and metastatic RCC patients, in which the tumor burden is elevated, and the immune microenvironment is strongly immunosuppressive. In contrast, personalized vaccines are administered as an adjuvant therapy following surgery, in a population with lower tumor burden, which likely improves efficacy. It should be emphasized that this was a single-arm, nine-patient phase I study without a control group, and the absence of recurrence, while encouraging, cannot be definitively attributed to the vaccine alone. This may suggest that the application of tumor vaccines should first focus on identifying available neoantigens, and secondly, the timing of administration should primarily aim for post-surgical prevention and adjuvant therapy, rather than attempting to salvage advanced patients. Especially in light of the fact that IO+TKI therapies have already established their position as first-line treatments, tumor vaccines in advanced RCC should complement and synergize rather than compete with these treatments.

#### 2.1.3. Oncolytic Viruses

Oncolytic viruses constitute a group of viruses that preferentially infect and destroy malignant cells, exerting dual anticancer effects. The virus replicates within tumor cells and causes cell lysis, directly killing the tumor cells. It also directly targets the mechanism of insufficient antigen release caused by non-immunogenic death of tumor cells and induces immunogenic death. Tumor antigens and virus-derived DAMP released after lysis activate an innate immune response that promotes dendritic cell maturation and antigen cross-presentation [27]. Therefore, this strategy not only directly induces tumor cell lysis, but amplifies anti-tumor immune responses by enhancing antigen release and presentation. Many challenges remain, however, for the clinical application of this strategy, including the limited penetration of the virus into tumor tissue, the short duration of effect, and the possibility that the host antiviral immune response may impair efficacy [28]. Additionally, the effectiveness of oncolytic viruses is constrained by the immunosuppressive tumor microenvironment, and monotherapy frequently fails to elicit a durable immune response.

In the field of RCC, multiple oncolytic virus strains are presently under investigation in preclinical and clinical studies, encompassing multiple types including poxvirus, measles virus, adenovirus, coxsackievirus, and even SARS-CoV-2. Systemic injection of oncolytic poxvirus can inhibit the growth of primary tumors and pulmonary metastases in metastatic RCC by remodeling the tumor microenvironment; the engineered Edmonston strain of measles virus shows enhanced anti-tumor effects against RCC cells; oncolytic adenoviruses delivering dual-strand small interfering RNA targeting Ki67 and hTERT genes have been shown to effectively induce apoptosis in RCC cells in vitro and in nude mouse models; synergistic effects have also been demonstrated with the combination of sunitinib and coxsackievirus [29,30,31,32,33]. In 2025, a report described a case of a stage IV RCC patient achieving complete remission after multiple intratumoral injections of oncolytic viruses, including Newcastle disease virus, type III coxsackievirus, and vaccinia virus. This remission lasted for 35 months without serious adverse events, representing the first reported case of complete remission in advanced RCC mediated by oncolytic viruses [34]. Although this case warrants attention and demonstrates the potential of oncolytic virotherapy, it represents an exceptional case rather than representative evidence, and its generalizability to the broader RCC population remains unknown. Currently, several clinical trials of oncolytic viruses in RCC are ongoing. A phase I clinical trial (NCT07431840) is assessing the safety of the oncolytic virus OTS-412 in combination with atezolizumab in advanced solid malignancies, including RCC. Another phase II trial (NCT07218692) is assessing the efficacy of oncolytic virus RP2 (sturlimogene erparepvec) combined with tivantinib in patients with metastatic RCC who have experienced progression following immunotherapy. From these studies, it is evident that oncolytic viruses represent a biologically intriguing strategy for RCC, though the current clinical evidence is largely confined to preclinical models, early-phase trials, and isolated case reports. Whether oncolytic viruses can deliver consistent clinical benefit in RCC nevertheless remains to be demonstrated in larger controlled trials. The efficacy of a single virus strain is still limited, so an oncolytic virus should be used as an activator and amplification agent of immune circulation in the treatment of RCC. How to optimize the viral vector and the combination therapy strategy with ICI or TKI is a key research direction for the future.

#### 2.1.4. Radiation-Induced Immunogenic Cell Death

Radiation therapy has been traditionally thought to primarily kill tumor cells by directly damaging their DNA. However, recent studies have shown that radiation, particularly stereotactic body radiation therapy (SBRT) with specific dosages and fractionation, can induce immunogenic cell death. High-dose radiation can cause tumor cell death in a short period of time and expose new tumor antigens, making the irradiated tumor cells act similarly to tumor vaccines after radiation exposure [35]. Simultaneously, DAMPs released during tumor cell death, including ATP, HMGB1, and heat shock proteins, can effectively promote dendritic cells to recognize and capture antigens, which are then presented in draining lymph nodes [36]. Moreover, SBRT can enhance the activity of immune agents by normalizing microvessels to relieve intratumoral hypoxia, improving drug delivery, and altering the immunosuppressive tumor microenvironment [35]. Therefore, radiation therapy plays a dual role in enhancing antigen release and improving the immune microenvironment, providing a theoretical basis for its combination with immunotherapy. Its mechanism is highly similar to that of oncolytic viruses; both induce immunogenic death to address the problem of insufficient antigen release. The patient populations for these strategies largely overlap, but SBRT has a slight advantage in the competition because of more mature evidence, more standardized operation, and a wider range of application.

RCC has long been considered a tumor that is resistant to radiation therapy, but this view is now being challenged [37]. With the advancement of stereotactic body radiotherapy technology, high-dose radiation can be accurately focused on tumor lesions while protecting the surrounding normal tissues to the greatest extent [35]. Recent evidence shows that SBRT for kidney tumors achieves a long-term local control rate of approximately 95%, with limited toxicity and minimal impact on renal function [38]. These favorable outcomes, however, reflect local disease control rather than systemic immunologic or survival benefit. SBRT has similar efficacy to cytoreductive nephrectomy, but it also has the advantages of non-invasive treatment and the ability to treat inoperable lesions due to tumor size or location [39]. In the current clinical application, this technique is mainly used for local control in oligometastatic patients, and synergistic efficacy is explored in combination with immunotherapy. Radiation-induced antigen release can promote the activation of T cells, mainly targeting antigen release, and partly improving immune rejection. ICIs can further reverse T-cell exhaustion, and together, these treatments synergistically enhance different phases of the immune cycle. Currently, several prospective studies exploring this combined strategy are underway.

Within this section, personalized neoantigen vaccines constitute one of the most promising therapeutic strategies, although their value in advanced RCC has yet to be confirmed. Oncolytic viruses and SBRT are not valuable as standalone treatments but rather in combination with ICIs or TKIs. Overall, there is no established first-line strategy targeting the antigen release phase, which underscores the fact that, in advanced RCC, simply addressing upstream issues without addressing downstream problems such as T-cell exhaustion and immune rejection will not effectively suppress the tumor.

### 2.2. Insufficient T-Cell Activation and Expansion

#### 2.2.1. Mechanistic Basis

Full activation of naive T cells occurs in two steps. They are initiated when the T-cell receptor recognizes the antigenic peptide–MHC complex and mediated by the binding of costimulatory molecules on antigen-presenting cells to their corresponding receptors on T cells [40]. CD28 is one of the most important costimulatory receptors, which can activate the downstream PI3K-AKT signaling pathway after binding to CD80/CD86 on the surface of antigen-presenting cells, and promote the proliferation, survival and effector function differentiation of T cells [41]. When the costimulatory signal is absent or insufficient, T cells can enter a dysfunctional state, that is, the initiation is impaired and the expansion is insufficient, and they cannot effectively perform anti-tumor immune functions [42]. Therefore, the integrity of co-stimulatory signaling is a key step connecting antigen presentation with T-cell effector functions.

Defects in the costimulatory signal are one of the important mechanisms of T-cell priming disorder in RCC. Tumor cells often lack key co-stimulatory molecules such as B7-1 (CD80), which limits their ability to effectively activate T cells [43]. Meanwhile, co-stimulatory molecules like B7-H1 (PD-L1), which are typically expressed only on macrophage lineage cells under normal physiological conditions, are abnormally highly expressed on RCC tumor cells [44]. By engaging PD-1 on T cells, B7-H1 transmits inhibitory signals that have been demonstrated to compromise T-cell function and induce apoptosis [44]. Studies have demonstrated that high expression of B7-H1 on RCC and tumor-infiltrating lymphocytes is significantly linked to tumor aggressiveness, and compared with patients exhibiting low B7-H1 expression, those with high expression have a 4.5-fold greater risk of death from RCC [42].

Immune suppressive cells represent another barrier to effective T-cell activation. Regulatory T cells (Tregs) are the most critical suppressive cell population. Tregs can continuously display elevated levels of CTLA-4, using this key molecule to suppress immune responses by competitively binding to CD80/CD86 on APCs, effectively blocking the CD28-mediated co-stimulatory signal and inducing the downregulation of CD80/CD86 expression on DCs [45]. CTLA-4 exhibits markedly greater affinity for CD80/CD86 than CD28, giving Tregs an advantage in competing for co-stimulatory molecules, thereby suppressing effector T-cell activation. Studies have demonstrated that the degree of Treg infiltration in RCC tissues is closely correlated with tumor stage, treatment resistance, and poor prognosis. After depleting Tregs, anti-tumor immune effects induced by vaccines and other methods in cancer patients significantly improve [46]. In addition to Tregs, MDSCs and TAMs also promote immune suppression and are important components of the tumor hypoxic microenvironment [47]. These cells not only promote tumor cell survival and proliferation by enhancing angiogenesis [48], but also recruit Tregs through the secretion of chemokines such as CCL17, CCL22, and immunosuppressive cytokines such as IL-10 and TGF-β, interfering with T-cell metabolic adaptation and functional maintenance, further inhibiting T-cell activation and expansion. They also secrete various growth factors to resist apoptosis stimulation [49]. These cells can secrete immunosuppressive cytokines like IL-10, TGF-β, and chemokines (such as CCL17, CCL22), recruit Tregs, and disrupt T-cell function, further suppressing T-cell activation and expansion [50]. Among these mechanisms, targeting costimulatory receptor antibody defects has been successfully translated into valuable therapeutic approaches. Therapies targeting MDSCs and TAMs, although supported by strong preclinical theory, have not yet been translated into approved therapies for kidney cancer.

#### 2.2.2. Cytokines

Cytokines, as critical mediators of immune responses, are pivotal in the development of immunotherapy for RCC. Molecules such as IL-2, interferon-α, and GM-CSF exert anti-tumor effects by activating effector cells, promoting antigen presentation and facilitating infiltration of immune cells [3]. In the immune cycle, cytokines primarily function by promoting T-cell activation and expansion, thus enhancing the anti-tumor immune response. By bypassing the need for co-stimulatory signals, cytokine therapy addresses the problem of insufficient T-cell activation due to the absence of co-stimulatory signals. Among them, IL-2 has the most widespread and representative clinical application.

High-dose IL-2 was approved by the U.S. Food and Drug Administration (FDA) in 1992, becoming the first immunotherapy for metastatic RCC. Its mechanism of action involves the activation of lymphocytes, including inducing natural killer (NK) cells and T cells to differentiate into lymphokine-activated killer cells, which show effective cytotoxic activity against various tumor cells while protecting normal tissues [3]. Fyfe et al. demonstrated that approximately 7% of metastatic RCC patients receiving high-dose IL-2 achieved long-lasting complete remission. The subsequent NCT00554515 clinical trial further confirmed that high-dose IL-2 can induce long-lasting remission and extend survival in patients across different risk categories [51]. However, the clinical use of IL-2 is constrained by considerable toxicity, such as vascular leak syndrome, hypotension, and multi-organ failure, which leads to severe adverse effects [52]. Interferon-α has been assessed in various studies, showing moderate benefits for progression-free survival and overall survival, but its clinical utility is limited by influenza-like symptoms, fatigue, and neuropsychiatric adverse effects [53]. Overall, while cytokine therapy occasionally leads to long-term remission, a substantial number of patients do not show significant improvement, and many experience severe adverse reactions. As a result, this therapy has gradually been replaced by emerging therapies such as targeted treatments and immune checkpoint inhibitors. Some investigations nonetheless suggest that IL-2 remains essential in certain subgroups of patients and is often used in combination with newer drugs [54].

Given the limitations of traditional IL-2, the development of modified cytokines has become a research hotspot. Interleukin-15 (IL-15) shares the β and γ chains of the IL-2 receptor but does not bind to the IL-2 receptor α chain. As a result, IL-15 selectively activates effector CD8+ T cells and NK cells, and maintains CD8+ memory T cells, potentially making it more suitable for cancer therapy than IL-2 [55]. Currently, IL-15 has entered clinical trials for RCC (NCT01021059) to assess its safety and efficacy [55]. However, studies investigating cytokine effects on normal epithelial and malignant cells remain limited, and further exploration is needed [56].

#### 2.2.3. Co-Stimulatory Receptor Agonists

One of the emerging research directions to address the issue of co-stimulatory signal deficiencies in renal cancer is the use of co-stimulatory receptor agonists, which are commonly presented in the form of antibodies. Unlike the non-specific activation induced by IL-2, these agonists mainly enhance T-cell activation, proliferation, and effector functions by mimicking the second signal provided by antigen-presenting cells. Theoretically, they do not activate non-specific immune cells and specifically target the “signal amplification” phase following T-cell initiation. Commonly studied targets include OX40, 4-1BB, GITR and ICOS, among others [40]. Additionally, novel antibody engineering techniques, such as bispecific antibodies, have provided new avenues for the precise regulation of co-stimulatory signals [57].

OX40 (CD134) is mainly expressed on activated T cells, such as the surface of Tregs, and its agonists can promote the proliferation of effector T cells, the formation of memory T cells, and inhibit Treg function, thereby helping to reduce Treg numbers [58]. The first phase clinical trial of OX40 yielded promising results [40]. In combination with other immunomodulators, OX40 exhibited good safety and tolerability but did not show significant efficacy [59,60]. The main mechanisms behind its failure are thought to include the transient expression dynamics of OX40, reactivation of regulatory T cells, metabolic inhibition, and insufficient FcγR-mediated crosslinking [61]. 4-1BB (CD137) is predominantly found on activated CD8+ T cells and NK cells, enhancing T-cell effector functions and survival [40]. Early 4-1BB agonists, such as urelumab and utomilumab, were limited in clinical application due to dose-limiting hepatotoxicity and very limited efficacy. Next-generation 4-1BB-targeting agents are currently under investigation to determine whether improved safety profiles can translate into clinical benefit [62]. GITR is expressed on both effector T cells and regulatory T cells [40], and its agonists primarily function by inhibiting T-cell apoptosis induced by TCR stimulation and altering the stability of Tregs to suppress their activity [63].

Furthermore, bispecific antibodies, by simultaneously targeting T cells and tumor antigens, enable the spatial coupling of co-stimulatory signals with tumor recognition, thus enhancing the specificity of immune activation [64]. A typical example in renal cancer is the chimeric bispecific G250/anti-CD3 monoclonal antibody, which has shown promise as an effective adjuvant in advanced renal cancer treated with IL-2 [65].

#### 2.2.4. Adoptive Cell Therapy (ACT)

ACT is a type of cellular therapy in which immune cells are isolated from the patient, expanded ex vivo, and subsequently reinfused into the same patient [66]. Thus, it can bypass the initiation and expansion of T cells in vivo, and solve the problems such as insufficient number or functional defects of T cells in patients. Depending on the source and processing of the cells, ACT includes chimeric antigen receptor T-cell therapy (CAR-T), tumor-infiltrating lymphocyte (TIL) therapy, cytokine-induced killer cell (CIK) therapy, and lymphokine-activated killer cell (LAK) therapy [67]. In recent years, clinical investigations of ACT in renal cancer have become increasingly active, with treatment strategies evolving from non-specific expansion to gene-engineered modifications.

Tumor-infiltrating lymphocyte therapy is the traditional form of ACT, in which lymphocytes are isolated from resected tumor tissue, non-specifically expanded ex vivo, and then reinfused into the patient [66]. This approach has been examined in renal carcinoma for over two decades, with a phase III clinical study published in 1999 demonstrating no meaningful differences in objective response rate or survival between TIL therapy combined with IL-2 and IL-2 alone [68]. More recently, new-generation TIL products are undergoing early clinical trials in solid tumors, utilizing optimized screening strategies, such as proteomic platforms to identify new antigen-reactive T cells for expansion.

Chimeric antigen receptor T-cell therapy is currently a hot research area within ACT, using genetic engineering to enable T cells to engineer synthetic receptors that bind specific tumor antigens, thereby allowing T cells to recognize and eliminate malignant cells in a major histocompatibility complex-restricted fashion [3]. However, in solid tumors like renal cancer, CAR-T therapy faces two major challenges: target selection and suppression within the tumor microenvironment [2]. Clinical trials of CAR-T for renal cancer primarily target CD70, CAIX, and other antigens, with several early-stage studies ongoing [2]. Moreover, chimeric antigen receptor natural killer (CAR-NK) cells and CAR-NK T cells, with their intrinsic immediate tumor recognition and cytotoxic abilities, along with lower adverse reaction risks, are seen as promising additions and future directions in the field of renal cancer cell therapy [67,69].

CIK and LAK cells, another form of adoptive cell therapy, are derived from an effector cell population with a broad spectrum of killing activity by cytokine stimulation in vitro [70]. CIK therapy has shown an objective response rate of approximately 25% in advanced renal cancer, which is one of the most effective strategies in each subtype [70]. However, its efficacy is still limited, and the most important challenge is the physical barrier and immunosuppression of the tumor microenvironment, as well as the loss and escape of tumor antigens. Therefore, more research has focused on the combination of other treatment methods.

In general, the clinical application of adoptive cell therapy in renal cell carcinoma is still in the early stage of exploration, showing limited objective response rate but good safety. Current evidence should be viewed as hypothesis-generating, and the translation of ACT into standard RCC care remains a long-term goal rather than a near-term prospect. No strategy in this segment holds a pivotal position in first-line treatment. Due to toxicity concerns, IL-2 has become a second-line therapy, co-stimulatory agonist monotherapy is ineffective, and ACT is still in the early stages of exploration. The absence of a solution in this phase highlights the importance of addressing microenvironmental suppression and T-cell exhaustion. Strategies targeting the T-cell initiation phase should also aim to supplement first-line treatments in cases of resistance or intolerance.

### 2.3. T-Cell Exhaustion and Functional Suppression

#### 2.3.1. Mechanistic Basis

T-cell exhaustion is one of the core mechanisms of tumor immune escape. In the tumor microenvironment of renal cancer, infiltrating CD8+ T cells exhibit typical features of exhaustion. In this microenvironment, persistent antigenic stimulation drives prolonged T-cell activation, resulting in a progressive reduction in effector function and ultimately a transition into a dysfunctional state termed T-cell exhaustion [71]. Moreover, the TILs specific to tumor antigens and MHC-restricted signals further promote this process [72,73]. In solid tumors such as renal cancer, T-cell exhaustion is often accompanied by a reduction in cytokine production, upregulation of inhibitory receptor expression, and the expression of immunosuppressive enzyme CD39 [71]. A key feature of this exhausted state is the upregulation of inhibitory receptors—referred to as immune checkpoints—such as PD-1, CTLA-4, LAG-3, and TIGIT. The expression of these receptors is closely linked to transcriptional regulatory programs associated with sustained antigenic stimulation [74]. Under physiological conditions, the expression of immune checkpoints helps to limit excessive immune responses and maintain immune homeostasis [71,75]. Once expressed within the tumor, these checkpoints mark T cells that have entered an exhausted state, thereby promoting disease progression [71]. This phenomenon also further supports the central role of local antigen persistence in driving depletion.

Inhibitors targeting the above-mentioned immune checkpoints partially reverse the exhausted state of T cells by blocking the binding of inhibitory receptors to their ligands [76], so as to achieve the purpose of anti-tumor activity. This therapy has been widely concerned and widely used in the clinical treatment of renal cancer.

#### 2.3.2. PD-1/PD-L1 Inhibitors

PD-1 is mainly expressed on the surface of activated T cells, and its ligand PD-L1 is widely expressed on tumor cells and antigen-presenting cells in the tumor microenvironment [77]. Their interaction transmits inhibitory signals to T cells and prevents their cytolytic activity. Antibodies that inhibit the PD-1/PD-L1 interaction can effectively restore T-cell effector functions. In addition to PD-L1, another ligand of PD-1, PD-L2, is more restrictively expressed, primarily on dendritic cells, macrophages, and B cells. However, recent studies suggest that simultaneous blockade of both PD-L2 and the PD-1/PD-L1 axis may further enhance tumor clearance, with the gut microbiome playing a regulatory role in this process [77].

Nivolumab is a fully humanized PD-1 monoclonal antibody and the first immune checkpoint inhibitor approved by the US FDA for clinical use in renal cancer. In 2006, Nivolumab was initially evaluated in a phase I single-dose escalation trial involving patients with RCC, marking the first PD-1 blockade in human history [78]. The subsequent phase III CheckMate-025 study also demonstrated the drug’s advantages with regard to both efficacy and safety [79].

Another PD-1 inhibitor approved by the US FDA for multiple cancer indications, including RCC, is pembrolizumab, a first-in-class humanized IgG4 monoclonal antibody [2]. In the phase II KEYNOTE-427 study, pembrolizumab as a first-line monotherapy for clear cell RCC patients achieved an objective response rate of 38.2%, with a complete response rate of 2.7%, further validating the therapeutic value of PD-1 blockade in renal cancer [79].

The success of PD-1 inhibitors lies in their direct targeting of the primary immune dysfunction in advanced renal cancer. Even if antigen presentation and T-cell initiation are impaired, partial reversal of the exhausted state of infiltrating T cells can still lead to clinical benefit. This is the fundamental advantage of PD-1 inhibitors compared to tumor vaccines and co-stimulatory receptor agonists.

In addition to the two PD-1 inhibitors mentioned above, PD-L1-targeting drugs have also gained a place in cancer treatment. Currently, clinically applied PD-L1 antibodies include atezolizumab, avelumab, and durvalumab [78]. These agents likewise produce antitumor activity through inhibition of the PD-1/PD-L1 signaling axis, although they are mainly used for other cancers, and their role in renal cancer treatment requires further research. Currently, the combination of avelumab and the tyrosine kinase inhibitor axitinib has received FDA approval as a first-line therapy for advanced RCC [80].

#### 2.3.3. CTLA-4 Inhibitors

Similar to PD-1, CTLA-4 is primarily expressed on the surface of regulatory T cells and activated T cells [81]. It competes with CD28 for interaction with CD80/CD86 on antigen-presenting cells, delivering inhibitory signals to T cells and restraining their overactivation and autoimmune injury. CTLA-4 inhibitors work by blocking this pathway, thereby enhancing the initial activation and expansion of T cells during the initiation phase and promoting anti-tumor immune responses [82]. Unlike the PD-1 pathway, which primarily modulates effector T cells in peripheral tissues, the CTLA-4 pathway mainly modulates T-cell activation within lymphoid organs. Ipilimumab is a fully humanized IgG1κ monoclonal antibody approved by the FDA, specifically targeting CTLA-4. However, its clinical use is often associated with significant immune-related adverse events [83]. Given the complementary mechanisms of action between CTLA-4 and PD-1 inhibitors—CTLA-4 enhances T-cell initiation, while PD-1 reverses T-cell exhaustion—the combination of the two has become an important therapeutic strategy [84]. In 2018, the US FDA approved nivolumab in combination with ipilimumab as a first-line treatment for high-risk, previously untreated metastatic RCC patients in accordance with the International Metastatic Renal Cell Carcinoma Database Consortium (IMDC) guidelines [79]. Additionally, a currently ongoing phase III study (NCT04736706) is evaluating the safety and efficacy of the combination of quavonlimab and pembrolizumab with lenvatinib versus pembrolizumab alone with lenvatinib [85]. The combination of CTLA-4 inhibitors with other ICIs, targeted therapies, and radiotherapy continues to be widely explored.

#### 2.3.4. LAG-3 Inhibitors

Lymphocyte Activation Gene-3 (LAG-3) is a transmembrane protein whose molecular structure is highly homologous to CD4 and can specifically recognize and interact with MHC class II molecules [86,87]. This molecule is present on multiple immune cell types, including activated and exhausted CD4+ and CD8+ T cells, as well as regulatory T cells [87,88]. LAG-3 suppresses T-cell proliferation, cytotoxic function, and homeostatic maintenance, thereby weakening immune responses against malignant cells and pathogens [89,90]. LAG-3 and PD-1 are frequently co-expressed on TILs, where they work synergistically to suppress T-cell function, contributing to the molecular foundation of T-cell exhaustion [76]. Therefore, the increased activation of alternative inhibitory pathways, such as LAG-3, after PD-1 inhibition is regarded as a major mechanism of resistance to immunotherapy, and dual blockade has been shown to strengthen antitumor immune activity [91]. Consequently, the FDA has authorized the first dual therapy combining anti-PD-1 and anti-LAG-3 agents [77].

Relatlimab is a specific humanized monoclonal antibody targeting LAG-3 [2]. In the field of RCC, relatlimab is currently undergoing phase II clinical trials. Preliminary results suggest that LAG-3 monotherapy or its combination with PD-1 inhibitors exhibits therapeutic potential for PD-1 inhibitor-resistant RCC patients with LAG-3 expression on their tumors [2]. Intravenous administration of relatlimab may induce adverse reactions including fatigue, skin rash, and joint pain [2], with overall safety characteristics similar to other immune checkpoint inhibitors. Several clinical studies assessing the efficacy of the combination of LAG-3 and PD-1 inhibitors are currently ongoing [92].

#### 2.3.5. Other Emerging ICIs

Besides PD-1, CTLA-4, and LAG-3, increasingly immune checkpoint targets such as TIGIT and TIM-3 have gained widespread attention in renal cancer immunotherapy research. These molecules are believed to represent compensatory pathways that mediate immune suppression following PD-1 blockade and are critical components of the immune resistance network. Consequently, they naturally form complementary relationships with PD-1 inhibitors, with their primary clinical application being in combination therapy with PD-1 inhibitors.

TIM-3 was initially discovered to be expressed on the surface of interferon-γ-producing CD4+ and CD8+ T cells [93,94]. Like PD-1, this molecule exerts a suppressive regulatory function in preserving immune homeostasis [77], but it is important to note that its function is context-dependent, and under certain conditions, it can also have stimulatory effects, making its role in immune regulation more complex [77]. TIGIT, a member of the CD28 immunoglobulin superfamily [95], is primarily expressed on natural killer cells, regulatory T cells, and activated T cells [96,97]. It plays a role in preventing autoimmune reactions by limiting excessive T-cell activation [98]. However, in RCC, evidence suggests that the overall expression level of TIGIT is relatively low [99]. In the tumor microenvironment, TIM-3 is often highly expressed on functionally exhausted T cells, and like TIGIT, TIM-3 is often co-expressed alongside PD-1 on tumor-infiltrating CD8+ T cells [100]. Studies have shown that combined blockade of PD-1 and TIM-3 is superior in efficacy in clearing viruses and enhancing tumor regression compared to blocking either receptor alone [86,101]. Similarly, simultaneous blockade of PD-1 and TIGIT is better than PD-1 inhibition alone in promoting tumor clearance [97,98].

Sabatolimab is a humanized monoclonal antibody directed against TIM-3 and is currently being evaluated in clinical trials across multiple solid malignancies as an immune myeloid drug [102,103]. The monotherapy efficacy of sabatolimab is limited, and thus its combination with PD-1 inhibitors is a key development direction. Tiragolumab is an IgG4κ monoclonal antibody directly targeting TIGIT, and is undergoing phase II clinical assessment in patients with metastatic RCC.

Overall, TIM-3 and TIGIT inhibitors appear to have limited efficacy as a monotherapy, and although preclinical models provide a rationale for their combination with PD-1 inhibitors, clinical evidence in renal cancer is still preliminary. Several trials are currently evaluating these combinations, and their therapeutic role, if any, awaits clarification from ongoing studies. In this context, PD-1/PD-L1 inhibitors are the most crucial therapeutic strategy, having established the foundation for immune therapy in RCC. Although CTLA-4 blockade shows limited efficacy as a single agent, its combination with PD-1 inhibitors has positioned it as a first-line treatment option for patients with intermediate- and high-risk disease. The future clinical positioning of LAG-3, TIGIT and TIM-3 inhibitors should be aimed at overcoming adaptive resistance to PD-1 inhibitors. The success of drugs targeting this phase highlights that “reversing T cell exhaustion” is the most direct and effective step in activating the anti-tumor immune cycle in renal cancer, which is the fundamental reason why ICIs (rather than tumor vaccines or cytokines) have become the cornerstone of RCC immunotherapy.

### 2.4. Immune Exclusion in the Tumor Microenvironment

#### 2.4.1. Mechanistic Basis

The tumor microenvironment (TME) of RCC exhibits unique immune suppressive characteristics, which are manifested in its high degree of vascularization and the accumulation of immune-suppressive cells. These features create a complex network that hinders immune cell infiltration and function. This immune-exclusion characteristic is primarily attributed to the tumor’s distinct molecular pathology. About 90% of clear cell renal carcinomas have inactivating mutations in the VHL gene, leading to the accumulation of hypoxia-inducible factor (HIF) and overexpression of vascular endothelial growth factor (VEGF) [104]. VEGF is not only a key angiogenic factor, but also a powerful immunomodulatory molecule. It forms a physical barrier by inducing abnormal angiogenesis, limiting the infiltration of effector T cells, inhibiting antigen presentation and promoting the recruitment and expansion of immunosuppressive cells [105,106]. The latter mechanism is particularly crucial as it directly facilitates the mobilization of myeloid-derived suppressor cells (MDSCs) from the bone marrow to the periphery, and drives tumor-associated macrophages (TAMs) towards immune-suppressive M2 polarization [106].

Metabolic dysregulation within the TME further exacerbates immune suppression. Tumor cells heavily rely on aerobic glycolysis, consuming large amounts of glucose and producing lactic acid, which acidifies the TME and suppresses T-cell proliferation and effector function [107,108]. Indoleamine 2,3-dioxygenase (IDO), abundantly expressed in malignant cells and TAMs, depletes tryptophan and produces kynurenine, which induces T-cell apoptosis and promotes the differentiation of regulatory T cells [109]. Hypoxia, as a central feature of the RCC TME, activates the adenosine pathway. Adenosine, when binding to the A2A receptor on T cells, delivers potent immunosuppressive signals [110]. Additionally, hypoxia can amplify these effects through the HIF pathway, further driving VEGF expression and promoting the conversion of MDSCs and TAMs to immunosuppressive phenotypes [111,112].

As key components of the TME and primary executors of the above-mentioned suppressive mechanisms, MDSCs and TAMs have been described in Section 2.2.1, and their roles in immune modulation, metabolic competition, and T-cell function inhibition will not be further elaborated here. Taken together, the VHL-HIF-VEGF axis represents the most clinically validated mechanism in renal cancer TME, with approved TKIs and HIF-2α inhibitors directly targeting the VHL-HIF-VEGF axis. Metabolic mechanisms, such as IDO and adenosine pathways, although supported by preclinical evidence, have yet to demonstrate clinical benefit in renal cancer based on negative clinical trials of IDO inhibitors.

#### 2.4.2. IO+TKI Combination Therapy

Given the multi-layered suppression of the tumor microenvironment, a single intervention strategy often proves insufficient. Currently, the clinical application of IO+TKI combination therapy, which targets the VEGF pathway to improve immune cell infiltration while simultaneously activating effector T-cell cytotoxicity, has become the first-line treatment for advanced metastatic renal cell carcinoma [113].

Initially developed as anti-angiogenesis agents, TKIs have since been recognized for their broad immune-modulatory effects, reshaping the TME on multiple levels [114], thus creating favorable conditions for ICIs to exert their effects. The TKI-mediated remodeling of the TME primarily focuses on regulating tumor growth and angiogenesis. By inhibiting VEGF receptors (VEGFR), TKIs normalize abnormal tumor vasculature [115], reduce vascular permeability, and improve blood flow, thereby facilitating effector T-cell infiltration into the tumor. Along with vascular normalization, tumor hypoxia is alleviated, further weakening the immune-suppressive TME. Additionally, various TKIs can reduce the number of Tregs and MDSCs [116].

Several phase III clinical trials have confirmed the efficacy of the IO–TKI combination as a first-line treatment for advanced RCC, with different TKIs displaying unique therapeutic profiles due to differences in their target spectra (Table 1).

Pembrolizumab combined with axitinib (KEYNOTE-426 study) was the first regimen to demonstrate the efficacy of IO+TKI combination therapy. Axitinib, a highly selective VEGFR inhibitor, primarily regulates immune modulation via the VEGF pathway [120]. The study demonstrated that combination therapy significantly prolonged overall survival (OS) and progression-free survival (PFS) relative to sunitinib alone, yielding an objective response rate (ORR) of 59% and a complete response (CR) rate of 5.8% [117]. Furthermore, the five-year follow-up analysis revealed a higher oral gastric response rate (41.9%) and a PFS of 15.1 months, further validating its therapeutic advantage [67].

Nivolumab combined with cabozantinib (CheckMate 9ER study) exemplifies the immune-modulatory benefits of multi-target TKIs. In addition to VEGFR inhibition, cabozantinib also targets MET, AXL, RET, and others [121], providing unique capabilities to directly combat MDSCs and promote M1 polarization. The study found that the combination therapy significantly outperformed sunitinib in both PFS and OS, with an ORR of 55.7% and a CR rate of 8.0% [118]. Three-year follow-up data demonstrated sustained therapeutic advantages, reflected in a higher median PFS (16.6 months) and OS [67].

Pembrolizumab combined with lenvatinib (CLEAR study) represents the combination of a broad-spectrum TKI with an ICI. Lenvatinib targets VEGFR1-3, FGFR1-4, PDGFR, KIT, and RET, making it one of the TKIs with the widest target spectrum [122]. The study showed that the combination therapy significantly improved PFS (median 23.9 months) and OS compared to sunitinib, with an ORR of 71% and a CR rate of 16.1% [119], giving it some of the best reported efficacy data for a first-line treatment of advanced RCC. Subsequent follow-up results from the CLEAR trial [123] and a phase Ib/II KEYNOTE-146 study [124] further supported its potential as a first-line treatment for advanced RCC.

**Figure 2:**
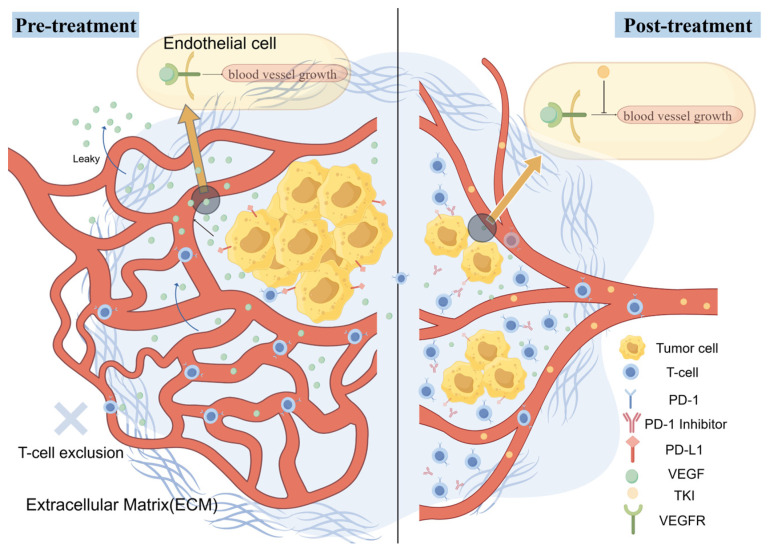
Synergistic mechanisms of IO+TKI combination therapy in RCC. This figure adopts a left-right comparison format to contrast the RCC tumor microenvironment before and after IO+TKI combination therapy. Before treatment: Tumor cells secrete VEGF, which acts on VEGFR on vascular endothelial cells, leading to abnormal, tortuous, and leaky vessels. These abnormal vessels block CD8+ T-cell extravasation. After treatment: TKIs enter vascular endothelial cells and inhibit VEGFR kinase activity, normalizing the vasculature. Normalized vessels allow CD8+ T cells to extravasate and infiltrate the tumor tissue. ICIs, exemplified by PD-1 inhibitors, block PD-1/PD-L1 interaction, reversing T-cell exhaustion and restoring cytotoxic function. TKIs overcome T-cell infiltration barriers, while ICIs restore T-cell function, together achieving synergistic antitumor immunity. Abbreviations: VEGFR, vascular endothelial growth factor receptor; VEGF, vascular endothelial growth factor; TKI, tyrosine kinase inhibitor; PD-L1, programmed death-ligand 1; PD-1, programmed cell death protein 1.

The IO+TKI combination therapy is not only the most important strategy for this phase but also the primary approach in current RCC immunotherapy. Its success lies in its simultaneous targeting of two core elements in the RCC immune cycle. On one hand, TKIs normalize the tumor vasculature via VEGFR inhibition, overcoming the physical barrier to T-cell infiltration and enhancing immune cell penetration into the tumor. On the other hand, ICIs reverse the exhausted state of infiltrating T cells, restoring their effector functions. These therapies complement each other (Figure 2). Furthermore, certain TKIs have independent immune-modulatory effects, such as decreasing the levels of MDSCs and Tregs, and promoting M1 polarization of TAMs, thereby creating further conditions for ICIs to act. It is the simultaneous intervention in both the immune-exclusion and T-cell dysfunction mechanisms within the immune cycle that allows IO+TKI therapy to significantly outperform any single-mechanism strategy. Additionally, the VHL-HIF-VEGF axis is a central oncogenic pathway in ccRCC, and TKIs directly inhibit this pathway while providing dual anti-tumor and immune-modulatory effects. In contrast, strategies like tumor vaccines and co-stimulatory agonists do not directly target RCC’s driver genes, and their efficacy is dependent on the patient’s endogenous immune state, placing them at a competitive disadvantage.

## 3. Major Limitations and Clinical Challenges

### 3.1. Drug Resistance

**Figure 3:**
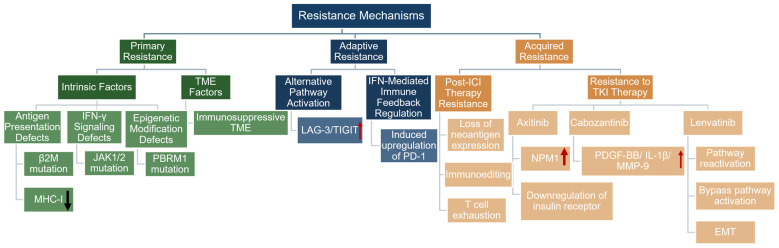
Mechanisms of therapeutic resistance in RCC. The figure categorizes resistance mechanisms into: (1) Primary resistance, involving intrinsic factors (antigen presentation, IFN-γ signaling, and *PBRM1* mutations) and TME-driven suppression; (2) adaptive resistance, characterized by alternative pathway activation (e.g., LAG-3/TIGIT) and IFN-mediated immune feedback; and (3) acquired resistance, occurring post-ICI therapy (immunoediting/exhaustion) or during TKI treatment (Axitinib, Cabozantinib, Lenvatinib) through bypass pathway activation and EMT. Red upward arrows indicate upregulation; black downward arrows indicate downregulation. Abbreviations: ICI, immune checkpoint inhibitor; EMT, epithelial–mesenchymal transition; IFN, interferon; MHC, major histocompatibility complex; JAK, Janus kinase; IL, interleukin; MMP, matrix metalloproteinase; NPM1, nucleophosmin 1; PDGF, platelet-derived growth factor; PD-1, programmed cell death protein 1; TME, tumor microenvironment; TKI, tyrosine kinase inhibitor.

As with other therapeutic modalities, resistance to immunotherapy in RCC remains a significant clinical challenge, with the most prominent area of investigation being resistance to ICIs. Resistance to ICIs in RCC can be categorized into three categories: primary resistance, adaptive resistance, and acquired resistance [125] (Figure 3). Primary resistance refers to the lack of any response to initial treatment, where the tumor does not react to immune checkpoint inhibitors; adaptive resistance refers to tumors that initially respond to immunotherapy but subsequently escape through dynamic adjustments to immune-suppressive pathways; acquired resistance refers to cases where patients achieve objective remission or disease stabilization following initial treatment but later experience disease progression during ongoing therapy [3,126]. In RCC, approximately 20–30% of patients exhibit primary resistance to PD-1/PD-L1 inhibitors as a monotherapy, and even with the combination of IO+TKI therapy, a significant proportion of patients fail to achieve objective responses [126].

RCC displays unique immune resistance characteristics: although tumor tissues are rich in TILs, these cells are often in a functionally exhausted state, marked by elevated expression of inhibitory receptors and impaired effector function. Additionally, RCC has a relatively low tumor mutational burden (TMB), a scarcity of B cells and tertiary lymphoid structures, and an accumulation of immune-suppressive myeloid cells [126], all of which contribute to the complex foundation of immunotherapy resistance [127].

Mechanistically, resistance arises from a combination of factors such as defects in antigen presentation, an immune-suppressive microenvironment, and the activation of alternative immune-suppressive pathways. Intrinsic tumor factors include defects in antigen presentation mechanisms, such as mutations in the β2-microglobulin gene leading to MHC-I assembly defects, which prevent tumor cells from being recognized by T cells [128]; abnormalities in interferon signaling pathways, such as JAK1/2 mutations that make tumor cells unresponsive to interferon-γ signaling [129]; and epigenetic modifications. PBRM1, a commonly mutated gene in RCC, has attracted considerable attention regarding its correlation with ICI efficacy. Recent studies have shown that PBRM1 mutations are closely linked to progression and drug sensitivity in ccRCC [130], and PBRM1 knockdown can improve the effectiveness of anti-PD-1 immunotherapy in RCC [131]. Tumor microenvironment factors primarily manifest as insufficient immune cell infiltration and an immune-suppressive environment, leading to what is termed “cold” tumors [3]. Recent transcriptomic analyses have shown that tumors from patients with primary resistance to nivolumab combined with ipilimumab treatment exhibit significantly reduced expression of immune checkpoint genes such as CTLA-4, TIGIT, and PD-1, suggesting that a lack of immune activity in the microenvironment is closely associated with dual immune resistance [132]. Furthermore, studies on the novel resistance marker KPNA2 reveal the impact of the microenvironment. High expression of KPNA2, despite an increase in tumor-infiltrating lymphocytes, is associated with reduced granzyme B expression in CD8+ T cells, indicating functional impairment. This is accompanied by an enrichment of Tregs and a reduction in M1 macrophages, forming an immune-suppressive microenvironment that leads to resistance to both TKI and immunotherapy [133].

Adaptive resistance often involves the upregulation of compensatory immune checkpoint molecules. Preclinical research has demonstrated that following PD-1 inhibition, tumor-infiltrating T cells can upregulate the expression of other inhibitory receptors such as LAG-3 and TIGIT, bypassing the blockade of the PD-1/PD-L1 axis [134]. Similar phenomena have been observed in RCC patients, where some patients who initially respond to PD-1 inhibitors show significantly increased expression of LAG-3 or TIGIT upon tumor progression. Moreover, interferon-γ signaling, while activating anti-tumor immunity, can also induce tumor cells to express PD-L1 and other immune-suppressive molecules, forming a negative feedback loop that contributes to adaptive resistance [135].

Acquired resistance mechanisms are more complex and are typically associated with the lack of neoantigen expression, immune editing, or T-cell exhaustion [3,136]. After ICI administration, clonal selection occurs within the tumor, favoring the survival of cells with low immunogenicity or reduced neoantigen expression. Additionally, in the context of IO+TKI combination therapy or monotherapy with TKIs, specific resistance mechanisms to TKIs may arise. For example, axitinib resistance is associated with upregulation of nucleophosmin 1 (NPM1) or reduced insulin receptor levels [137], notably, NPM1 has recently been identified as a key mediator in ALDH9A1-regulated AKT signaling and lipid metabolism reprogramming in ccRCC [138], further linking metabolic dysregulation to therapy resistance; cabozantinib resistance may be mediated by the secretion of non-VEGF angiogenesis factors such as PDGF-BB, IL-1β, MMP-9, and IL-8 [137], while lenvatinib resistance is linked to the reactivation of previously targeted pathways, activation of alternative pathways, and epithelial-to-mesenchymal transition (EMT) [137]. These interconnected mechanisms complicate the progression of disease following combination therapy.

Not all of the above resistance mechanisms are equally translational from a clinical perspective. Following IO+TKI failure, compensatory upregulation of LAG-3 and TIGIT, as well as IFN-γ-mediated PD-L1 re-induction, represent the most clinically relevant categories, providing a direct rationale for next-generation checkpoint combination therapy or continued PD-1 pathway blockade. In contrast, tumor-intrinsic events such as JAK1/2 mutations, β2-microglobulin deficiency, and neoantigen loss through immunoediting, as well as TKI resistance mechanisms involving alternative angiogenic pathways such as PDGF-BB, IL-8, or epithelial–mesenchymal transition, are mechanistically well defined but are only conceptual in the absence of strategies to combat them. Other factors, including low TMB, PBRM1 mutation status, KPNA2 overexpression, and scarcity of B cells and tertiary lymphoid structures, contribute to a broader resistance profile but remain primarily relevant observations that do not yet guide clinical decision making. These distinctions highlight the gap between mechanistic understanding and clinical application, underscoring the need for continued translational research to bridge the two.

### 3.2. Impact of Immune-Related Adverse Events (irAEs)

While ICIs enhance anti-tumor immune responses, they also disrupt the balance of immune tolerance, potentially affecting virtually every organ system [79] and causing a wide range of irAEs. The pathophysiology of these events remains incompletely understood. Over 70 different types of irAEs with varying degrees of severity have been reported, presenting unique challenges for clinical management [76].

IrAEs typically occur within weeks to months of administration but can manifest at any time during treatment or even after therapy has been discontinued [139,140]. Therefore, regular monitoring of patient conditions is essential. Different ICI regimens are associated with distinct profiles of irAEs. Fatigue, pruritus, and rashes are the most common adverse events [79], but they are typically mild and do not require treatment discontinuation. Other irAEs include gastrointestinal issues such as diarrhea and colitis, endocrine dysfunctions like hypothyroidism, adrenal insufficiency, pituitary inflammation, and acute kidney injury, predominantly due to acute interstitial nephritis [141], as well as more severe but less common events such as myocarditis, myositis, and life-threatening myasthenia gravis. If not promptly managed, these complications can lead to severe consequences [79].

In a multi-center retrospective analysis of 356 advanced RCC patients treated with ipilimumab and nivolumab, the overall irAE incidence was 76.4%, with 35.4% of cases involving severe adverse events (grade 3 or above). Additionally, 27.3% of patients required high-dose corticosteroid intervention [142]. The incidence of irAEs leading to treatment discontinuation and death has also raised concerns in pivotal phase III trials. In the KEYNOTE-426 study, the pembrolizumab plus axitinib group reported 11 treatment-related deaths, including fatalities due to myasthenia gravis, myocarditis, cardiac arrest, pneumonia, and pulmonary embolism [117]; the JAVELIN Renal 101 study also reported three treatment-related deaths in the avelumab plus axitinib group, caused by myocarditis, sudden death, and necrotizing pancreatitis [143]. These observations underscore the importance of early recognition and intervention for irAEs.

Moreover, a growing body of evidence indicates that the occurrence of non-lethal irAEs is positively correlated with the efficacy of immune therapy [76,144]. However, the appearance of irAEs is not a requisite for effective response, as some patients who experience irAEs may still maintain durable therapeutic responses and extended treatment-free intervals [79]. This phenomenon suggests that irAEs could serve as clinical biomarkers of effective immune system activation, and patients with these adverse reactions may achieve long-term benefits. Nevertheless, additional in-depth investigation is required to explore this hypothesis.

The management of irAEs typically follows a graded approach [145]. Mild toxicities generally do not require discontinuation of treatment and can be managed with close monitoring or symptomatic treatment. Moderate to severe toxicities necessitate the suspension of immunotherapy and the administration of corticosteroids [145]. For steroid-resistant cases, other immunosuppressive agents such as infliximab may be considered [145]. Multidisciplinary collaboration is also crucial in the management of irAEs, involving oncologists, dermatologists, gastroenterologists, pulmonologists, and endocrinologists, among others [146,147].

## 4. Biomarkers for Immunotherapy in RCC

Despite the central role of immunotherapy in advanced renal cancer, few molecular biomarkers other than the IMDC clinical risk model have entered routine practice to guide treatment decisions. The markers discussed below cover prognosis, prediction, resistance, and treatment sequencing (Table 2).

Among prognostic indicators, IMDC risk stratification remains the basis for evaluating patient outcomes [148]. Several readily available laboratory parameters, including elevated neutrophil-to-lymphocyte ratio (NLR) and C-reactive protein (CRP), were associated with shorter overall survival [149], while at the histological level, sarcomatoid differentiation predicted particularly aggressive disease [150]. The intrinsic features of the tumor that drive a poor prognosis do not always predict treatment failure; however, as discussed below, sarcomatoid renal cell carcinoma is also one of the histologic types most responsive to ICIs. At the molecular level, PD-L1 expression on tumor and immune cells is associated with cancer-specific mortality [42], and Treg infiltration has a similar prognostic role [46], but neither has formally become a clinically feasible assay.

The search for predictive markers for immunotherapy benefits mainly focuses on tumor genomics. PBRM1 loss-of-function mutations are found in about 40% of ccRCC tumors, and PBRM1 loss-of-function mutations have become one of the most consistent correlates of response to PD-1 blockade treatment [151]. Because the tumor mutational burden in clear cell renal cell carcinoma is usually low, markers that are helpful for other malignancies are less useful in renal cancer and usually have limited predictive value [126]. Furthermore, the presence of tertiary lymphoid structures of a tissue-local immune response has been associated with improved outcomes during PD-1 inhibitor therapy [152], and spatial HLA signatures capturing tumor immune structures have shown promise in predicting clinical responses [153]. Sarcomatoid renal cell carcinoma (SRCC), despite its poor prognosis, has shown high ICI sensitivity in various trials [150] and remains a worthy direction of exploration, although the relevant biology remains unclear.

Resistance to immunotherapy has been discussed in detail in Section 3.1. From a biomarker perspective, several molecular features have emerged as potential indicators of different resistance phenotypes. β2-microglobulin mutations [128] and JAK1/2 alterations [129] are associated with primary resistance, whereas compensatory upregulation of LAG-3 and TIGIT [134] and overexpression of KPNA2 [133] are associated with acquired resistance. Alternatively, TGF-β-driven T-cell rejection may lead to an immune cold phenotype refractory to checkpoint blockade. Although these markers offer mechanistic possibilities, their clinical utility for prospectively identifying resistant patients remains untested.

Biomarkers for treatment sequencing remain the least developed category, as prospective data directly comparing different sequences in RCC are scarce. In current practice, only the IMDC model informs first-line selection. Beyond clinical risk stratification, retrospective analyses have suggested that PBRM1 status and immune gene expression signatures may associate with differential benefit from specific regimens [132,151], and molecular classifications distinguishing angiogenic from immune-inflamed subtypes could eventually refine treatment selection [154]. Dynamic markers such as circulating tumor DNA are also under investigation, with early data suggesting that on-treatment ctDNA changes correlate with response to TKIs plus ICIs [150], though these approaches remain investigational.

In summary, the field of biomarkers for RCC has expanded substantially, but further efforts are needed to translate it into clinical practice, with prospective validation in biomarker-stratified trials.

## 5. Future Directions

While IO+TKI combinations have redefined first-line care for advanced RCC, primary resistance occurs in about 20–30% of patients, and acquired resistance eventually develops in most patients. It suggests to us that existing therapies, while a good starting point, are far from an end point. Future explorations in immunotherapy, particularly multi-immune checkpoint combinations, IO + metabolic modulation, and personalized immunotherapy, show great promise.

PD-1+CTLA-4 dual immunotherapy (nivolumab + ipilimumab) has achieved good results in renal cancer, and has become one of the first-line options for intermediate and high-risk patients. This validates the feasibility of targeting multiple immune checkpoints simultaneously. On this basis, other ICI combination regimens with complementary mechanisms, such as PD-1+LAG-3 and PD-1+TIGIT, are becoming hot future research directions. The core reason is that the compensatory upregulation of inhibitory receptors such as LAG-3 and TIGIT after PD-1 blockade is the core mechanism of acquired drug resistance, which cannot be solved by simply increasing the dose of PD-1 or replacing antibodies, and these compensatory pathways must be targeted at the same time. From the perspective of clinical positioning, the core value of this new combination strategy is mainly that for patients with disease progression after PD-1 treatment and tumors expressing LAG-3 or TIGIT, the combination of corresponding inhibitors is the most direct rescue plan. The feasibility and safety of using these novel combinations to prevent drug resistance at the time of initial treatment also warrant further exploration. Despite limited progress in exploring new immune checkpoints in recent years, future research should focus on how best to utilize existing targets. One approach would be multi-target network blockade, simultaneously targeting PD-1, LAG-3, TIGIT and other molecules to break the compensatory network. Another approach could involve sequential blockade, using corresponding inhibitors based on the upregulation of different checkpoints during resistance evolution. Regardless of the approach, the key lies in personalized treatment, namely selecting the appropriate combination based on profiling receptor expression in patient tumor tissues.

The potential of IO + metabolic modulation arises from a deeper understanding of RCC’s metabolic characteristics. While IO+TKI effectively inhibits the VEGF pathway, the metabolic dysregulation in RCC’s tumor microenvironment is multifaceted. Lactate accumulation, IDO-mediated tryptophan depletion, and hypoxia-induced activation of the adenosine pathway all form a deeper immune-suppressive network that TKI cannot fully resolve. In this context, HIF-2α inhibitors like belzutifan have emerged. HIF-2α is a key molecule in the core RCC driver pathway (VHL-HIF-VEGF axis). Belzutifan inhibits HIF-2α, blocking both VEGF expression and hypoxia-induced metabolic reprogramming upstream, and has synergistic potential with ICIs. It is particularly applicable to ccRCC patients who have already been treated with PD-1 or PD-L1 checkpoint inhibitors and VEGF-TKIs [155]. In the future, the focus may not be on discovering more metabolic targets, as many phase III trials of IDO inhibitors and A2A receptor antagonists have failed or shown limited efficacy [156]. The true direction should be to fully exploit the synergistic effects of existing targets, creating an “immune + anti-vascular + metabolic modulation” three-dimensional approach, while developing biomarkers to identify patients who are most likely to benefit from metabolic modulation for precise applications.

The success of personalized neoantigen vaccines in adjuvant treatment of RCC (with a median follow-up of 40.2 months and no recurrences in 9 high-risk post-surgical patients) suggests that designing treatment plans based on a patient’s unique mutation profile is a promising direction. Personalized immunotherapy is becoming one of the key trends in future treatments. However, personalized immunotherapy goes beyond vaccines and encompasses CAR-T, TCR-T, and personalized TIL therapies. Future exploration in this field needs to address issues such as preparation cycles, cost, target selection, optimization of combination strategies, and patient selection. Additionally, the clinical implementation of personalized immunotherapy requires biomarker-driven precision stratification. Currently, the IMDC risk stratification system continues to serve as the core tool guiding treatment selection for advanced RCC patients [148]. The IO+TKI regimen is suitable for patients across all IMDC risk strata [157,158,159], while nivolumab + ipilimumab is only approved for intermediate- and high-risk patients [142]. This clinical stratification logic suggests that personalization should not only focus on molecular-level neoantigens or genetic mutations but should also be reflected in the rational application of clinical risk stratification. On this basis, PBRM1 mutations (found in approximately 40% of RCC patients) have been shown to be associated with improved ICI efficacy and could act as a potential biomarker to predict the benefit of PD-1 inhibitors [151]. Other factors, such as TMB and HLA typing, are also under exploration [153,160]. In the future, combining IMDC clinical stratification with molecular biomarkers could enable individualized, precise treatment. Overall, personalized immunotherapy represents a concrete manifestation of the precision oncology concept in RCC immunotherapy. Although still in its early stages, it represents the major future development direction in RCC treatment.

## 6. Discussion

This article uses the cancer-immunity cycle as a framework to examine the major strategies in renal cancer immunotherapy across four key stages: antigen release and presentation (tumor vaccines, oncolytic viruses, stereotactic body radiation therapy, SBRT), T-cell activation and expansion (cytokines, co-stimulatory receptor agonists, adoptive cell therapy), T-cell exhaustion reversal (PD-1/PD-L1 inhibitors, CTLA-4 inhibitors, LAG-3 inhibitors, and other immune checkpoint inhibitors [ICIs]), and tumor microenvironment immune evasion (IO+TKI combination therapy).

Among these strategies, significant differences in efficacy are evident across different approaches. Strategies targeting antigen release and presentation have limited overall prospects. Phase III studies of peptide vaccines and dendritic cell vaccines have both failed, as these approaches address only the upstream mechanisms without resolving the downstream issues of T-cell exhaustion and immune evasion, making it difficult to achieve durable effects in advanced renal cancer. Oncolytic viruses and SBRT, as “in situ vaccines,” have some value, but their monotherapy effects are limited, and they are more often used as adjunctive therapies in combination treatments. Strategies targeting T-cell activation and expansion have similarly failed to become mainstream: IL-2 has been relegated to second-line treatment due to toxicity concerns, co-stimulatory receptor agonists show minimal efficacy as monotherapies, and adoptive cell therapies are still in the early stages of exploration. In contrast, strategies focused on reversing T-cell exhaustion have shown markedly different results: PD-1/PD-L1 inhibitors have become the cornerstone of immunotherapy, CTLA-4 inhibitors have limited efficacy alone but have established the dual immune standard when combined with PD-1 inhibitors in intermediate- and high-risk patients, and new ICIs like LAG-3 and TIGIT have shown potential in a post-resistance setting. To date, the combination therapy of IO+TKI in the immune rejection of the tumor microenvironment is the most effective strategy. With the dual advantages of intervening in the infiltration disorder and functional inhibition of T cells at the same time, it has become the standard first-line treatment for advanced RCC (Table 3).

To provide a practical framework for the reader, Table 4 stratifies the immunotherapeutic strategies discussed in this review according to their current level of clinical evidence. At one end of the spectrum, IO+TKI combinations and dual immune checkpoint inhibition (nivolumab plus ipilimumab) represent the only phase III-validated, practice-changing strategies and constitute the current standard of care for advanced RCC. At the other end, co-stimulatory agonists, TIL therapy, SBRT as an immune primer, and metabolic pathway targeting remain at the preclinical or early exploratory stage—supported by biological rationale but lacking clinical validation in RCC. Between these two poles, several strategies, including personalized neoantigen vaccines, next-generation checkpoint inhibitors (LAG-3, TIM-3, TIGIT), HIF-2α inhibitors, CAR-T therapy, and oncolytic viruses, are under active early-phase clinical investigation, with their definitive roles yet to be established. Notably, peptide and dendritic cell vaccines (IMA901, AGS-003) were investigated in phase III trials but failed to demonstrate clinical benefit, underscoring the challenges of translating vaccine strategies into advanced RCC. This stratification aims to distinguish what is truly ready for clinical application from what remains investigational or has been investigated without success.

## 7. Conclusions

Tracing the evolution of RCC immunotherapy, it becomes evident that progress is defined by our increasingly granular grasp of the cancer-immunity cycle. We are witnessing a fundamental pivot: moving beyond the blunt immune activation seen in the cytokine era toward the nuanced, multi-modal regulation of the IO+TKI standard, and eventually, metabolic-integrated and personalized regimens. The next frontier lies not just in adding more checkpoints, but in decoding the dynamic crosstalk within immune networks and the metabolic microenvironment’s remodeling. Realizing this transition requires moving past empirical “cocktail” therapies; instead, we must prioritize biomarker-driven precision stratification and adaptive frameworks that adjust in real time under dynamic monitoring. Only then can we bridge the gap between generalized combination therapy and truly tailored clinical decision-making.

## Figures and Tables

**Table 1:** Key phase III trials of first-line IO+TKI combinations in advanced renal cell carcinoma.

Regimen	Trial	ORR	CR Rate	Median PFS	Approved Population	Countries	References
Pembrolizumab + Axitinib	KEYNOTE-426	59%	5.8%	15.1 months	All IMDC risk groups	Multinational	[67,117]
Nivolumab + Cabozantinib	CheckMate 9ER	55.7%	8.0%	16.6 months	All IMDC risk groups	Multinational	[67,118]
Pembrolizumab + Lenvatinib	CLEAR	71%	16.1%	23.9 months	All IMDC risk groups	Multinational	[119]

**Table 2:** Key biomarkers in RCC immunotherapy.

Category	Biomarker	Immune Cycle Association	References
Prognostic	IMDC risk model	No	[148]
	NLR	No	[149]
	CRP	No	[149]
	Sarcomatoid differentiation	No	[150]
	PD-L1 expression	Yes	[42]
	Treg infiltration	Yes	[46]
Predictive	PBRM1 mutation	No	[151]
	Tertiary lymphoid structures (TLS)	Yes	[152]
	HLA spatial signature	Yes	[153]
	Sarcomatoid differentiation	No	[150]
Resistance	β2-microglobulin mutation	Yes	[128]
	JAK1/2 alterations	Yes	[129]
	LAG-3/TIGIT upregulation	Yes	[134]
	KPNA2 overexpression	No	[133]
Sequencing	Immune gene expression signatures	Yes	[132,151]
	Angiogenic vs. immune-inflamed subtypes	No	[154]
	ctDNA dynamics	No	[150]

**Table 3:** Summary of immunotherapeutic strategies in renal cell carcinoma.

Phase	Strategy	Representative Agent	Clinical Status	Clinical Positioning	Countries	References
Antigen release and presentation	Peptide vaccine	IMA901	Phase III failed; development terminated	Discontinued (phase III failed to improve OS)	Multinational	[22]
	Dendritic cell vaccine	AGS-003	Phase III terminated early for futility	Discontinued (phase III terminated early for futility)	US	[23]
	Personalized neoantigen vaccine	Personalized neoantigen vaccine	Phase I (NCT02950766)	Adjuvant therapy	US	[26]
	Oncolytic virus	Poxvirus, measles virus, adenovirus, coxsackievirus	Phase I/II ongoing	Investigational, combination with ICI	US, Germany	[29,30,31,32,33]
	SBRT	Stereotactic body radiation therapy	Clinical application	Combination with ICI	——	[38,39]
T-cell activation and expansion	Cytokine	High-dose IL-2	Approved	Second-line therapy (limited by toxicity)	US	[51,52,54]
	Co-stimulatory agonists	OX40, 4-1BB agonists	Phase I/II	Investigational	Multinational	[40,62]
	Adoptive cell therapy	TIL, CAR-T, CIK	Early clinical	Investigational	Multinational	[2,67]
T-cell exhaustion reversal	PD-1 inhibitor	Nivolumab, Pembrolizumab	Approved	Cornerstone of immunotherapy	Multinational	[78,79]
	CTLA-4 inhibitor	Ipilimumab	Approved	First-line combination	Multinational	[79]
	LAG-3 inhibitor	Relatlimab	Phase II ongoing	Post-resistance exploration	Multinational	[2]
	TIM-3 inhibitor	Sabatolimab	Phase II ongoing	Investigational	Multinational	[102,103]
Tumor microenvironment immune exclusion	IO+TKI	Pembrolizumab + Axitinib, Nivolumab + Cabozantinib, Pembrolizumab + Lenvatinib	Phase III approved	First-line standard of care	Multinational	[117,118,119]
	HIF-2α inhibitor	Belzutifan	Approved	Future combination direction	Multinational	[155]

**Table 4:** Stratification of immunotherapeutic strategies in RCC by level of evidence.

Evidence Level	Strategies
Approved Standard Therapies	IO+TKI combinations (Pembrolizumab + Axitinib; Nivolumab + Cabozantinib; Pembrolizumab + Lenvatinib)
	Nivolumab + Ipilimumab (PD-1 + CTLA-4 dual ICI)
	PD-1 inhibitors (Nivolumab, Pembrolizumab)
Phase III Investigated but Negative or Discontinued	Peptide vaccines (IMA901)
	Dendritic cell vaccines (AGS-003)
	High-dose IL-2
Early-Stage Clinical Investigation	Personalized neoantigen vaccines
	LAG-3 inhibitors (Relatlimab)
	TIM-3 inhibitors (Sabatolimab)
	TIGIT inhibitors (Tiragolumab)
	HIF-2α inhibitors (Belzutifan)
	CAR-T therapy
	Oncolytic viruses
Preclinical Concepts	Co-stimulatory agonists (OX40, 4-1BB, GITR)
	TIL therapy
	SBRT as immune primer
	Metabolic/IDO/Adenosine pathway targeting

## Data Availability

No new data were created or analyzed in this study. Data sharing is not applicable to this article.

## Abbreviations

The following abbreviations are used in this manuscript:

ACT	Adoptive Cell Therapy
AKT	Protein Kinase B
ATP	Adenosine Triphosphate
BAP1	BRCA1-Associated Protein 1
CAR-NK	Chimeric Antigen Receptor Natural Killer Cell
CAR-T	Chimeric Antigen Receptor T Cell
CCL17	Chemokine (C-C motif) Ligand 17
CCL22	Chemokine (C-C motif) Ligand 22
ccRCC	Clear Cell Renal Cell Carcinoma
CD28	Cluster of Differentiation 28
CD40L	CD40 Ligand
CD56	Cluster of Differentiation 56
CD8	Cluster of Differentiation 8
CD80	Cluster of Differentiation 80
CD86	Cluster of Differentiation 86
CIK	Cytokine-Induced Killer Cell
CR	Complete Response
CRP	C-Reactive Protein
CTLA-4	Cytotoxic T-Lymphocyte-Associated Protein 4
ctDNA	Circulating Tumor DNA
DAMP	Damage-Associated Molecular Pattern
DC	Dendritic Cell
DNA	Deoxyribonucleic Acid
EMT	Epithelial–Mesenchymal Transition
FDA	U.S. Food and Drug Administration
FGFR	Fibroblast Growth Factor Receptor
GITR	Glucocorticoid-Induced TNFR-Related Protein
GM-CSF	Granulocyte-Macrophage Colony-Stimulating Factor
HER2	Human Epidermal Growth Factor Receptor 2
HIF	Hypoxia-Inducible Factor
HLA	Human Leukocyte Antigen
HMGB1	High-Mobility Group Box 1
hTERT	Human Telomerase Reverse Transcriptase
ICAM1	Intercellular Adhesion Molecule 1
ICI	Immune Checkpoint Inhibitor
ICOS	Inducible T-Cell Co-Stimulator
IDO	Indoleamine 2,3-Dioxygenase
IFN	Interferon
Ig	Immunoglobulin
IL	Interleukin
IL-10	Interleukin-10
IL-15	Interleukin-15
IL-2	Interleukin-2
IL-6	Interleukin-6
IL-8	Interleukin-8
IMDC	International Metastatic Renal Cell Carcinoma Database Consortium
IO	Immuno-Oncology
ITIM	Immunoreceptor Tyrosine-Based Inhibition Motif
JAK	Janus Kinase
KPNA2	Karyopherin Subunit Alpha 2
LAG-3	Lymphocyte Activation Gene-3
LAK	Lymphokine-Activated Killer Cell
M-CSF	Macrophage Colony-Stimulating Factor
MDSC	Myeloid-Derived Suppressor Cell
MET	Mesenchymal–Epithelial Transition Factor
MHC	Major Histocompatibility Complex
MHC-I	Major Histocompatibility Complex Class I
MMP	Matrix Metalloproteinase
MUC1	Mucin 1
NK	Natural Killer Cell
NLR	Neutrophil-to-Lymphocyte Ratio
NPM1	Nucleophosmin 1
ORR	Objective Response Rate
OS	Overall Survival
OX40	Tumor Necrosis Factor Receptor Superfamily Member 4
PBRM1	Polybromo 1
PD-1	Programmed Cell Death Protein 1
PDGFR	Platelet-Derived Growth Factor Receptor
PD-L1	Programmed Death-Ligand 1
PD-L2	Programmed Death-Ligand 2
PFS	Progression-Free Survival
PI3K	Phosphoinositide 3-Kinase
RCC	Renal Cell Carcinoma
RET	Ret Proto-Oncogene
RNA	Ribonucleic Acid
SBRT	Stereotactic Body Radiation Therapy
SIGLEC-7	Sialic Acid-Binding Ig-Like Lectin 7
TAM	Tumor-Associated Macrophage
TCR	T-Cell Receptor
TGF-β	Transforming Growth Factor Beta
TIGIT	T-Cell Immunoreceptor with Ig and ITIM Domains
TIL	Tumor-Infiltrating Lymphocyte
TIM-3	T-Cell Immunoglobulin and Mucin Domain-Containing Protein 3
TKI	Tyrosine Kinase Inhibitor
TMB	Tumor Mutational Burden
TME	Tumor Microenvironment
TNF-α	Tumor Necrosis Factor Alpha
Treg	Regulatory T Cell
TSLP	Thymic Stromal Lymphopoietin
VEGF	Vascular Endothelial Growth Factor
VEGFR	Vascular Endothelial Growth Factor Receptor
VHL	Von Hippel–Lindau
